# Correction: Naphthylisoindolinone alkaloids: the first ring-contracted naphthylisoquinolines, from the tropical liana *Ancistrocladus abbreviatus*, with cytotoxic activity

**DOI:** 10.1039/d2ra90105f

**Published:** 2022-10-24

**Authors:** Shaimaa Fayez, Torsten Bruhn, Doris Feineis, Laurent Aké Assi, Prem Prakash Kushwaha, Shashank Kumar, Gerhard Bringmann

**Affiliations:** Institute of Organic Chemistry, University of Würzburg Am Hubland D-97074 Würzburg Germany bringman@chemie.uni-wuerzburg.de; Department of Pharmacognosy, Faculty of Pharmacy, Ain-Shams University Organization of African Unity Street 1 11566 Cairo Egypt; Federal Institute for Risk Assessment Max-Dohrn-Str. 8-10 D-10589 Berlin Germany; Centre National de Floristique, Université d’Abidjan, Conservatoire et Jardin Botanique Abidjan 08 Ivory Coast; Molecular Signaling & Drug Discovery Laboratory, Department of Biochemistry, Central University of Punjab Bathinda-151401 Punjab India

## Abstract

Correction for ‘Naphthylisoindolinone alkaloids: the first ring-contracted naphthylisoquinolines, from the tropical liana *Ancistrocladus abbreviatus*, with cytotoxic activity’ by Shaimaa Fayez *et al.*, *RSC Adv.*, 2022, **12**, 28916–28928, https://doi.org/10.1039/D2RA05758A.

The authors regret that the author affiliations were incorrectly shown in the original manuscript. The corrected list of affiliations is as shown above.

The Royal Society of Chemistry apologises for these errors and any consequent inconvenience to authors and readers.

## Supplementary Material

